# Supplementary material for: The importance of multiregional accounting for corporate carbon emissions
Source: Nat Commun. 2025 Dec 20;17:1014. doi: 10.1038/s41467-025-67759-5 (PMC12847884; doi:10.1038/s41467-025-67759-5)
Supplement: Supplementary file 1 — Supplementary Information [file 41467_2025_67759_MOESM1_ESM.pdf]

Supplementary Information for

The importance of multiregional accounting for corporate carbon emissions

Davis et al.

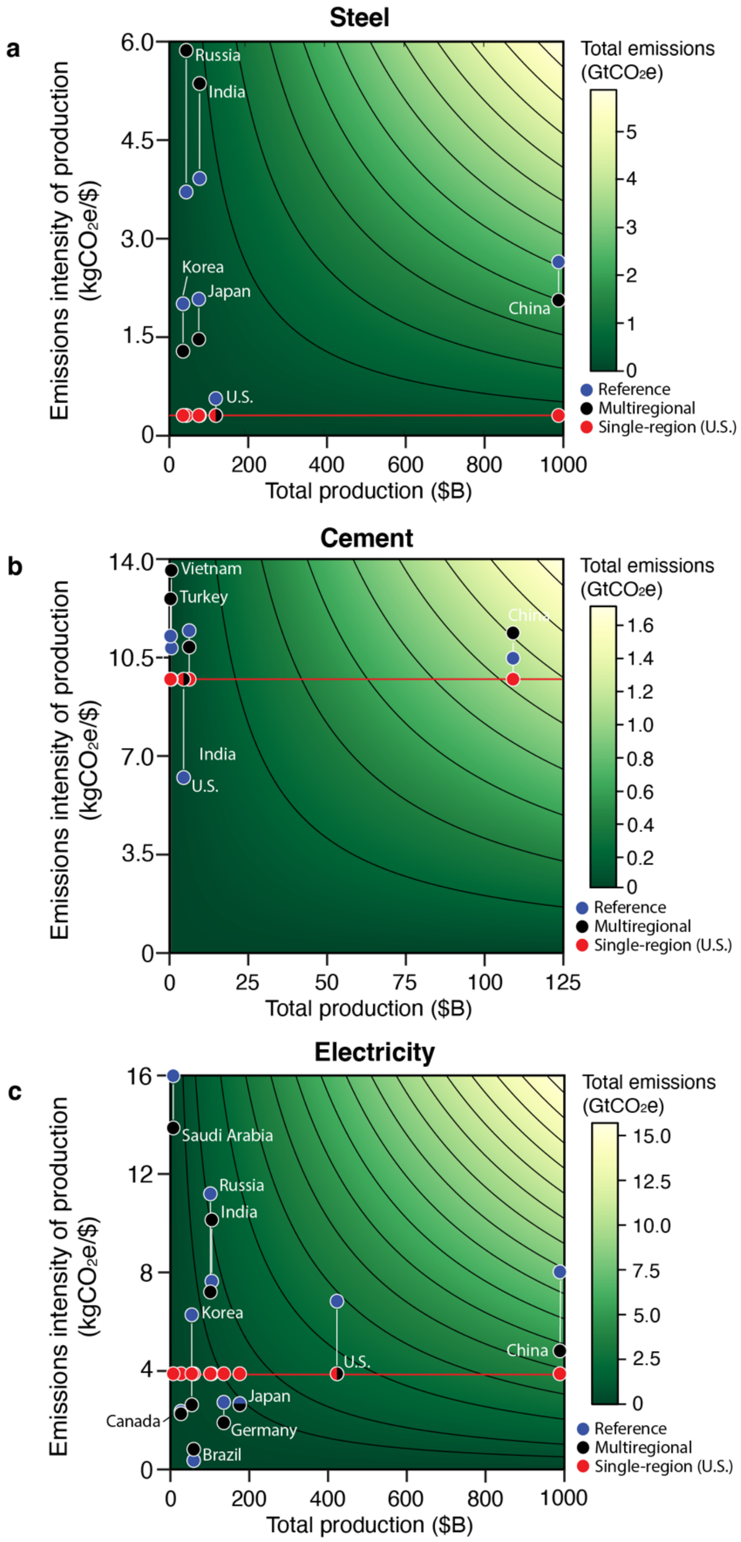

Supplementary Figure 1 | Comparison of key commodities' emissions intensity. Colored contours indicate country-level emissions related to steel (**a**), cement (**b**), and electricity (**c**) as the product of emissions per monetary unit produced (y-axis) and total production (x-axis). Points in each panel then show relevant values from our single-region (U.S.-based) input-output model (red circles), our multiregional model (CEDAv5.0, black circles), and reference values from published sources (blue circles, refs. *34-39*). To facilitate comparisons, reference and multiregional values are connected by vertical white lines, and a horizontal red line emphasizes the static single-region intensity. In many cases, the multiregional model estimates are closer to the reference values, which is particularly important in cases like China where total production of these commodities is very large.

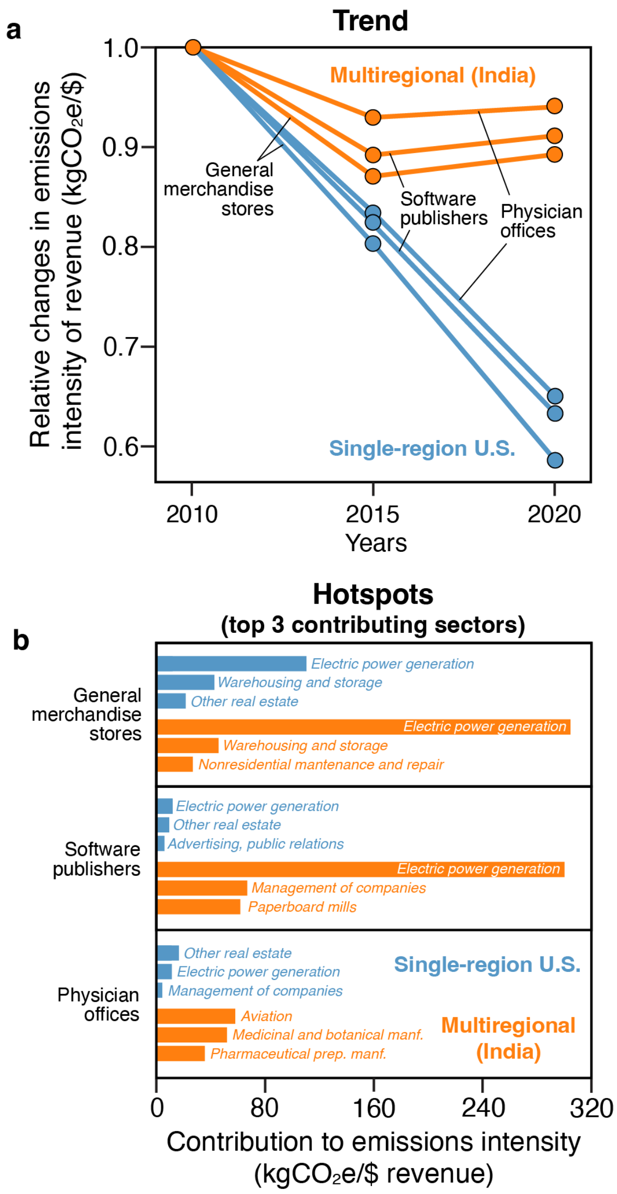

Supplementary Figure 2 | Estimated differences in Scope 3.1 emissions intensity of Indian sectors using single- and multi-regional EEIO models. In the three industry sectors of Indian companies that most commonly report Scope 3.1 emissions to CDP, trends in emissions intensity per unit revenue over time (**a**) and top 3 upstream hotspots (**b**), are substantially different when using a single-region (U.S.-based) input-output model (blue curves and bars) as compared to our multiregional model (CEDAv5.0, orange curves and bars).
